# The Management of Young Infants

**Published:** 1913-01-25

**Authors:** 


### MATERNITY AND MOTHERING.

(Inquiries and Correspondence are invited.)

### The Management of Young Infants.

Although the general outlines of the care and management of young babies, including the arrangement of their meals, have long been carefully studied, there are still disputed points as to which individual experience and prejudice dictate some latitude. And some of the hitherto accepted canons of the art, which seemed to be of rigid and established firmness, have lately been called in question by enterprising experimenters who have no veneration for any rule merely because it is ancient and greatly respected. It is still very doubtful how far these new ideas will gain acceptance and how far they are applicable to the ordinary run of babykind, which cannot command the services of an expert bacteriologist or milk at eighteenpence a pint. But, inasmuch as the results claimed are good, it certainly behoves those who undertake the care of their own or other people's children to be informed of the broad outlines of some of these novel theories.

### On a Desert Island.

Those who read a romance published a few years ago under the title of "The Blue Lagoon" will remember that the two castaways upon an uninhabited island found themselves possessed of a new-born infant, of whose needs they were both totally ignorant. Divining from its cries that the babe was hungry, they offered it milk out of a cocoanut, which it swallowed greedily and then incontinently vomited. Only by a chance contact did the mother discover that Nature had provided the proper nourishment, and the author remarked that the child knew more about infant feeding than its parents. The superiority of mother's milk over all other infant foods is, of course, unchallenged, notwithstanding some remarks by Dr. Eric Pritchard which were misinterpreted a few months ago and led to an amusing controversy in a nursing journal. Hitherto, the iconoclasts have not ventured to attack this position; their innovations have been chiefly in connection with artificial feeding.

In Mr. Stacpoole's romance, to which we have just referred, he proceeds to represent the shipwrecked parents as offering their baby from quite a tender age such additional food as bananas and similar products of their Pacific island. And he states that notwithstanding the horror with which the medical profession would have regarded this diet, the infant throve in quite a remarkable way. Whether Mr. Stacpoole was merely drawing a bow at a venture or whether he had heard of Budin's revolutionary plans of infant feeding we do not know; but at any rate his little shaft of irony landed not so very wide of its mark.

Supposing that for any reason, good or bad, it is decided to hand-feed a baby, Budin advises that after the end of the first month it should be given whole (cow's) milk, not the usual diluted mixture containing more water than milk that has hitherto been thought appropriate for infants at that tender age. Each meal will, of course, be much less in bulk than would be the case were a greatly diluted mixture being offered. Dr. Freeland, who advocates this system, says that the bottle containing the feed should be placed in boiling water for forty minutes, in order to ensure complete sterilisation; after the third month this period of immersion may be gradually shortened. He also decries the usual two-hourly interval between the meals of a newly-born baby. The infant's stomach does not get sufficient rest, he says, if food is given every two hours. Intervals of rest with an empty stomach are important factors in normal digestion; and at the Rotunda Hospital in Dublin babies have lately been fed at three-hourly intervals from birth with good results. In England Dr. Langmead is well known as an advocate of somewhat similar views. He gives undiluted and unboiled milk to which a few grains of sodium citrate have been added. Citrates, it is stated by one author, have lately been added to the composition of one of the best-known and least objectionable patent foods.

Children properly fed on whole milk thrive, put on firm healthy flesh, and gain in weight without becoming flabby or excessively fat. This much may be allowed; but it is also important that they will often suffer from constipation unless they are allowed drinks of plain (sterilised) water between meals. The stools are much bulkier than in breast-fed children, but it is claimed that there is much less tendency to flatulence and to posseting. It has to be recognised that many children take some little time to get accustomed to this diet, and that they are often fretful and apparently unhappy during the process of settling down to it. While admitting that as a rule the child does well, it may be questioned whether it does better than it would do on the usual system of modified cow's milk.

For babies under a month Dr. Freeland does not recommend whole milk, though some of the followers of Budin administer it right away from birth. He prefers (for a child which is not nursed at all by the mother) to start with a mixture of one part of milk to three parts barley water, with milk sugar. He gradually reduces the proportion of barley water without altering the total bulk of the meal, until at a month the child is having whole milk. Barley water contains starch, and the capacity of infants to digest starch is not developed at birth; it appears as a rule between the third and sixth month. The use of barley water is, however, defended on the ground that meals should not be entirely assimilated in either children or adults; a residue is necessary to promote healthy intestinal action. Eructations and distension are said not to occur on this starch-containing diet, provided always that the bowels are kept acting freely. This is not always so easy a matter as it sounds. Babies are notoriously prone to diarrhœa on little provocation; and they are also very often affected by constipation on no provocation at all. So starch in the dietary of quite tiny babies is still a controversial question.

